# The cytosolic DNA‐sensing cGAS–STING pathway in neurodegenerative diseases

**DOI:** 10.1111/cns.14671

**Published:** 2024-03-08

**Authors:** Xiaofeng Guo, Lin Yang, Jiawei Wang, You Wu, Yi Li, Lixia Du, Ling Li, Zongping Fang, Xijing Zhang

**Affiliations:** ^1^ Department of Critical Care Medicine, Xijing Hospital The Fourth Military Medical University China; ^2^ Department of Intensive Care Unit Joint Logistics Force No. 988 Hospital Zhengzhou China; ^3^ Department of Anesthesiology, Xijing Hospital Fourth Military Medical University Shaanxi China; ^4^ Translational Research Institute of Brain and Brain‐Like Intelligence, Shanghai Fourth People's Hospital, School of Medicine Tongji University Shanghai China

**Keywords:** cGAS, innate immune, neurodegenerative diseases, STING

## Abstract

**Background:**

With the widespread prevalence of neurodegenerative diseases (NDs) and high rates of mortality and disability, it is imminent to find accurate targets for intervention. There is growing evidence that neuroimmunity is pivotal in the pathology of NDs and that interventions targeting neuroimmunity hold great promise. Exogenous or dislocated nucleic acids activate the cytosolic DNA sensor cyclic GMP‐AMP synthase (cGAS), activating the stimulator of interferon genes (STING). The activated STING triggers innate immune responses and then the cGAS‐STING signaling pathway links abnormal nucleic acid sensing to the immune response. Recently, numerous studies have shown that neuroinflammation regulated by cGAS‐STING signaling plays an essential role in NDs.

**Aims:**

In this review, we summarized the mechanism of cGAS‐STING signaling in NDs and focused on inhibitors targeting cGAS‐STING.

**Conclusion:**

The cGAS‐STING signaling plays an important role in the pathogenesis of NDs. Inhibiting the cGAS‐STING signaling may provide new measures in the treatment of NDs.

## Graphical abstract

The cGAS‐STING pathway is anticipated to emerge as a novel therapeutic target for the management of neurodegenerative diseases.

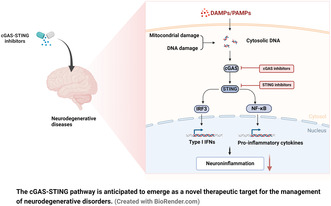

## 1 INTRODUCTION

Neurodegenerative diseases (NDs) are caused by loss of neurons and/or their myelin sheaths, which worsen over time and develop into functional impairment.^1^, ^2^ In most countries, the incidence of NDs increases with the extension of life expectancy. The pathogenesis of NDs may involve several aspects, such as oxidative stress, mitochondrial dysfunction, excitotoxicity, ER stress, axonal transport defects, neuroinflammation, and programmed cell death (PCD), which include apoptosis, autophagy‐dependent cell death, pyroptosis, necroptosis, and ferroptosis.^3^, ^4^, ^5^, ^6^ However, neuroinflammation and immunity play a central role in the pathogenesis of NDs.

The innate immune system is the first line of defense in mammals against the invasion of pathogenic microorganisms. It can recognize pathogen‐associated molecular patterns (PAMPs) through nucleic acid‐sensing pattern recognition receptors (PRRs) that promote elevated expression of type I interferons(IFN) and pro‐inflammatory factors.^7^, ^8^, ^9^ Cyclic GMP‐AMP synthase (cGAS) is the predominant sensor for invading microbial pathogens such as viruses or pathogens, while stimulator of interferon genes (STING) is a stimulator of interferon genes as an adaptor protein orchestrated by cGAS for host protection. The cGAS‐STING signaling pathway has an expanding role as an innate immune sensor in many pathological and physiological processes, including defense against microbial infection, antitumor immunity, autoimmunity, cellular senescence, and autophagy.^10^, ^11^, ^12^, ^13^ Recent studies have shown that STING binds directly to light chain 3 (Lc3) to induce non‐canonical autophagy, which clears cytosolic DNA to maintain a dynamic balance of STING function.^14^, ^15^ Meanwhile, STING‐induced apoptosis can also clear dying cells.^3^ Emerging evidence indicated that STING triggers neuroinflammation and various cell death pathways, including lysosome‐dependent cell death, pyroptosis, and necroptosis.^16^ Furthermore, much evidence has shown that the cGAS‐STING signaling pathway is critical in cancer, kidney diseases, diabetic cardiomyopathy, and NDs.^17^, ^18^, ^19^, ^20^

Above all, aberrant hyperactivation of the innate immune signaling pathway cGAS‐STING is essential to the pathological development of many neurodegenerative brain diseases. Therefore, small‐molecule modulators targeting cGAS‐STING are essential for the treatment of associated NDs. This review summarized the recent progress of cGAS‐STING signaling in NDs and the related modulators of pathway inhibition, which shed new light on the remedy for NDs.

## 2 THE cGAS‐STING SIGNALING PATHWAY

cGAS recognizes and is activated by DNA ligands, and then assembles into a dimer. Activated cGAS catalyzes the synthesis of cyclic 2′,3′‐cyclic guanosine monophosphate (cGAMP) from ATP and GTP.^21^ cGAMP will bind and activate an ER‐associated adaptor protein which is named STING, as mentioned above. Activated STING translocates from the endoplasmic reticulum to the intermediate compartment of the Golgi apparatus^22^ while recruiting and activating TANK binding kinase 1(TBK1).^23^, ^24^ In addition, activated TBK1 phosphorylates the transcription factor interferon regulatory factor 3 (IRF3) and promotes transcription of type I IFN.^25^ The cGAS‐STING signaling pathway also triggers a prominent pro‐inflammatory cytokine response through the activity of nuclear factor κB (NF‐κB) to enhance the release of interleukin‐6 (IL‐6) and tumor necrosis factor‐α (TNF‐α).^26^, ^27^

### 2.1 Activation of cGAS

The PRRs of host cells recognize PAMPs in different parts of the cell, which is the first step in triggering the innate immune response. cGAS belongs to the nucleotidyltransferase family members. The cGAS is a cytosolic DNA sensor that triggers the type I IFN pathway.^28^ Generally, cGAS is activated by pathogenic DNA and induces autoimmune responses. In addition, it senses various endogenous DNAs, such as microbial DNA, released mitochondrial DNA, extranuclear chromatin, cytosolic microbial DNA, and aberrant chromosomal DNA.^7^ However, not all DNAs can activate cGAS because the activation is strongly correlated with the length of dsDNA. Specifically, it is difficult to activate the signaling pathway if cGAS is limited or the DNA sequence is short and only initiated when longer stretches of dsDNA elicit a signal above a certain threshold.^7^, ^29^ Research demonstrates for the first time that DNA larger than 45 bp can stably activate cGAS.^30^ It should be noted that the lengths of DNA will trigger the activity of cGAS vary between different species, human and murine cGAS can be effectively activated by dsDNA longer than 45 and 20 bp, respectively.^31^, ^32^, ^33^

Upon cytosolic DNA binding, cGAS is activated by conformational transitions. The catalytic pocket of cGAS changes from closed to open, causing the formation of the cGAS‐DNA complex and catalyzing the generation of cGAMP.^34^ This process divides into two steps, cGAS uses ATP and GTP as substrates to transfer ATP onto the 2′‐OH of GTP to generate a canonical intermediate pppG(2′, 5′)pA,^35^ and then GTP is transferred onto the 3′‐OH of the adenosine nucleotide phosphate using pppG(2′‐5′)pA as a substrate.^7^ cGAMP is a second messenger that binds and activates STING, completing intracellular signal transduction and activating immune responses.

### 2.2 Activation of STING

STING is a multidomain transmembrane protein composed of an N‐terminal four transmembrane helical, a central ligand binding domain (LBD), and a C‐terminal tail (CTT) involved in binding TBK1. The domain LBD forms irregularly shaped dimers that are highly conserved and are important for STING to be transferred to perinuclear regions and activate downstream signaling pathways. Domain CTT is a linear signaling hub that can acquire modular motifs to easily adapt downstream immunity, which is primarily responsible for recruiting and activating TBK1 and IRF3.^36^ STING is localized to the endoplasmic reticulum membrane and partially to mitochondria and mitochondria‐associated membranes.^25^, ^37^ It is a central player in the innate immune response to nucleic acids (especially cytoplasmic dsDNA) and in the response to various pathogens. Excessive activation of STING may lead to autoimmune diseases such as systemic lupus erythematosus.^38^

When cGAMP binds and activates STING located in the endoplasmic reticulum, it leads to a change in STING dimer conformation.^25^ Dimeric STING is released from its anchored protein and subsequently transported to perinuclear structures through the endoplasmic reticulum Golgi intermediate compartment (ERGIC).^39^ In particular, in addition to cGAMP, endoplasmic reticulum stress, viral liposomes, and bacterial cyclic dinucleotide (CDN) are also able to activate sting and downstream signaling pathways directly as well.^40^

### 2.3 Intracellular signal transduction and immune response

A previous study has shown that the C‐terminal tail of STING contains a highly conserved PLPLRT/SD motif that mediates TBK1 recruitment and activation. Activated STING can recruit TBK1 through this motif to form STING‐TBK1 complex.^41^ TBK1 phosphorylates the serine residue of the pLxIS motif in the structural domain of CTT, making this motif a binding site for IRF3 and activating IRF3, which in turn promotes downstream IFN‐I transcription.^23^, ^42^

In addition, STING is not restricted to the type I IFN pathway, TBK1 also catalyzes the translocation of the transcription factor NF‐κB, which contains the p50 and p65 subunits and is essential for response to inflammatory stimuli.^3^ Research demonstrated that cGAS‐STING expression in HEK293T cells increased NF‐κB promoter activity by more than 100‐fold,^43^ and conversely, NF‐κB activation enhanced STING signaling by regulating microtubule‐mediated STING transport.^44^ During STING activation, TBK1 is redundant for IκB kinase (inhibitor of nuclear factor kappa‐B kinase, IKK) to promote NF‐κB release,^45^ which means activated STING can directly activate downstream IKK and release NF‐κB.^46^ A study also illustrated that IRF3 activation was highly dependent on TBK1 kinase activity, whereas NF‐κB sensitivity to TBK1/IKKε kinase was significantly reduced.^45^

Subsequently, activated IRF3, NF‐κB enters the nucleus to initiate downstream signals that directly or indirectly regulate the expression of type I IFN genes, interferon‐stimulated genes (ISG), and other inflammatory mediators, pro‐apoptotic genes, and chemokines.^46^, ^47^ Modifications include phosphorylation and ubiquitination of STING, which completes signal transduction, inhibits activity, and prevents overactivation of the natural immune response.

### 2.4 Non‐canonical cGAS‐STING signaling pathway

The non‐classical cGAS‐STING signaling pathway is not dependent on the activation of TBK1‐IRF3 and NF‐κB. It is mainly controlled by PKR‐like endoplasmic reticulum kinase (PERK) and eukaryotic initiation factor 2α (eIF2α). The activation of the STING‐PERK‐eIF2α pathway was shown to precede the activation of the STING‐TBK1‐IRF3 pathway. This cGAS‐STING‐PERK pathway regulates cap‐dependent messenger RNA translation, innate immune control of cellular senescence or organ fibrosis, and non‐canonical targeting or drug treatment of the cGAS‐STING pathway gene can alleviate pulmonary and renal fibrosis.^48^

## 3 REGULATION OF cGAS‐STING SIGNALING PATHWAY

The cGAS‐STING pathway, like many other signaling pathways, employs intricate regulatory mechanisms to maintain signal balance. The prevention of cytosolic DNA binding to cGAS by nuclease‐mediated degradation represents an effective mechanism for negative regulation. Additionally, post‐translational modification of signaling molecules plays a critical role in regulating pathway activity and serves as the underlying mechanism for many inhibitors' actions. Moreover, the cGAS‐STING pathway interacts with multiple signal pathways such as RIG‐I‐MAVS, thereby augmenting the complexity of its signal regulation and functionality.

### 3.1 Cellular nucleases

The recognition of cytosolic DNA by cGAS initiates the activation of the cGAS‐STING pathway, and thus, cellular elimination of such DNA is a crucial mechanism to prevent excessive pathway activation. The nucleases currently reported as candidates for fulfilling this role include DNaseII, three‐prime repair exonuclease 1 (TREX1), ANKLE1, and SAMHD1. DNaseII is predominantly localized in lysosomes and exhibits optimal activity under acidic conditions. It primarily functions to degrade DNA within endosomes and autophagosomes, thereby preventing its release into the cytoplasm.^49^ TREX1, also known as RNaseIII, is primarily to degrade mismatched DNA within cells and maintain cellular genetic stability. Abnormal functioning of TREX1 can result in the accumulation of DNA within cells.^50^ The recently discovered endonuclease ANKLE1 is localized to the midbody and plays a crucial role in managing aberrant chromatin bridges that arise during cell division, thereby preventing DNA damage and the accumulation of cytosolic dsDNA.^51^ SAMHD1, widely recognized as a dNTPase, can enhance the activity of the MRE11 enzyme and degrade nascent ssDNA at stalled replication forks, thereby impeding the accumulation of cytosolic ssDNA fragments.^52^ Dysfunction in any nuclease results in an abnormally elevated level of cytosolic DNA, subsequently leading to hyperactivation of the cGAS‐STING pathway.

### 3.2 Post‐translational modifications

Recent studies have shown that post‐translational modification of signaling molecules is another way of regulating cGAS‐STING pathway activity, and there are many inhibitors studied targeting this process. The most relevant post‐translational modifications reported to the cGAS‐STING pathway include ubiquitination, palmitoylation, and phosphorylation. We summarize the specific molecular media and effects in Table 1.

**TABLE 1:** The post‐translational modifications of cGAS‐STING pathway.

Post‐translational modifications	Mediator	Function	Effects	Reference
Ubiquitination	TRIM29 TRIM30α TRIM13	Induces the k48‐linked polyubiquitination of STING	Mediates the degradation of STING	Zhang et al. (2020) Wang et al. (2015) Li et al. (2022)
	TRIM56 TRIM41	Induces the monoubiquitination of cGAS	Increases the dimerization and DNA binding activity of cGAS	Yang et al. (2018) Liu et al. (2018)
	AMFR‐INSIG1	Induces the k27‐linked polyubiquitination of STING	Promotes the activition of STING	Wang et al. (2014)
Deubiquitination	USP29	Removes the K48‐linked polyubiquitination of cGAS	Reduces the degradation of cGAS	Zang et al. (2020)
	OTUD5 USP20 CYLD USP44	Removes the K48‐linked polyubiquitination of STING	Reduces the degradation of STING	Guo et al. (2021) Zhang et al. (2016) Zhang et al. (2018) Zhang et al. (2020)
	USP13	Removes the K27‐linked polyubiquitin chain of STING	Inhibits the activation of STING	Sun et al. (2017)
Palmitoylation	ZDHHC18	Mediates the palmitoylation of cGAS at C474	Inhibits the activation of cGAS	Shi et al. (2022)
	ZDHHC1	Mediates the palmitoylation of STING at Cys88/91	Promotes the activition of STING	Mukai et al. (2016) Takahashi et al. (2021)
Phosphorylation	EGFR	Mediates the phosphorylation of STING at Tyr245	Promotes the activition of STING	Wang et al. (2020)
	BLK2	Mediates the phosphorylation of cGAS at Y215	Enhances the cytosolic retention of cGAS	Liu et al. (2018)

### 3.3 Crosstalk between RIG‐I‐MAVS and cGAS‐STING pathway

In addition to DNA, RNA in the cytoplasm can also be detected by specific receptors, namely retinoic acid‐inducible gene I (RIG‐I) like receptors (RLRs), which serve as the primary surveillance system for cytoplasmic RNA.^53^, ^54^ Although distinct receptors are responsible for the perception of DNA and RNA, their downstream signals exhibit interdependence, establishing crosstalk between RIG‐I‐MAVS and cGAS‐STING pathway.^55^

The RLRs family comprises of three members, RIG‐I, melanoma differentiation‐associated protein 5 (MDA5), and laboratory of genetics and physiology 2 (LGP‐2).^54^ Among these, both RIG‐I and MDA5 have two amino‐terminal caspase activation and recruitment domains (CARDs) which mediate downstream signaling. LGP‐2 lacks CARDs and is thought to be involved in the regulation of RIG‐I and MAD5. When cytoplasmic RNA activates RIG‐I and MDA5, they undergo conformational changes that expose the CARDs domain. Subsequently, the interaction between CARD‐CARD recruits MAVS located on the mitochondrial outer membrane, which serves as the core adaptor proteins for RLR signaling. This leads to a series of signaling cascades that ultimately activate IRF3, IRF7, and NF‐κB, inducing the expression of type I IFN and other pro‐inflammatory cytokines.^53^

STING can participate in the immune response against RNA viruses as a downstream effector of RIG‐I. After stimulation by RNA viruses, poly (I:C), and 5′pppRNA, RIG‐I is activated to upregulate the expression of STING.^56^, ^57^, ^58^ Knockout of STING leads to decreased expression of IFN and other cytokines, resulting in an elevated intracellular RNA viral load.^56^ Yiliu et al. proposed an autocrine/paracrine theory to elucidate the underlying mechanism of RIGI‐induced STING expression. After activation of RIG‐I, type I IFN and TNF‐α are secreted leading to autocrine/paracrine activation of transcription factors STAT and NF‐κB that induce the expression of STING.^57^

The study conducted by Rebecca et al. initially proposed the involvement of MAVS in STING's recognition of DNA. Their experimental findings demonstrated that the absence of both STING and MAVS in macrophages resulted in a 99% reduction in the IFN response after 4 h of Poly dA: dT stimulation, whereas this reduction was not observed in macrophages lacking STING.^59^ Furthermore, TBK1 phosphorylation following DNA virus infection relies on the interaction between MAVS and TBK1. Upon stimulation with dsDNA transfection in HeLa cells, approximately 60% of p‐TBK1 was found to co‐localize with MAVS, and subsequent knockout of MAVS significantly decreased cytoplasmic DNA‐induced levels of p‐TBK1 and IFN‐β.^60^ G10 can serve as an activator for the STING‐IRF3 axis, promoting IFN production. However, G10‐induced secretion of IFN is greatly diminished when MAVS is absent.^61^ All these observations suggest that MAVS may play a role in regulating the activity of the cGAS‐STING pathway.

## 4 THE cGAS‐STING SIGNALING PATHWAY IN NDs

Efficient neuronal function in the central nervous system (CNS) is inextricably linked to the formation and maintenance of a highly controlled microenvironment by astrocytes and microglia, which regulate CNS inflammation by secreting a variety of cytokines and inflammatory mediators.^62^ Astrocytes can respond to inflammatory signals and promote inflammation, regulating multiple life processes of the nervous system in both physiological and pathological states. Nuclear transport of NF‐κB in astrocytes is triggered by pro‐inflammatory stimuli, such as TNF‐α, IL‐1B and IL‐17, ROS, phagocyte myelin, Toll‐like receptors, and other associated factors with CNS inflammation.^63^, ^64^ Microglia activation by viral or damaged mtDNA initiates the cGAS‐STING signaling pathway, which secretes IFNs that act on interferon A receptors (IFNARs) on neurons to trigger neuronal antiviral defense mechanisms.^65^ The study revealed for the first time that microglia and astrocytes exhibit high levels of cGAS protein expression after quiescence and activation, while these cell types also constitutively express the critical downstream cGAS adaptor protein, STING.^66^

The cGAS‐STING signaling pathway plays an important role in NDs. Recent studies on human CNS tissues have established that the cGAS‐STING pathway is activated in both endothelial and neuron cells in NDs.^67^ In particular, excessive activation of this pathway can lead to fatal neuroinflammation, which in turn causes NDs such as ischemic stroke, Alzheimer's disease (AD), Parkinson's disease (PD), Huntington's disease (HD), and amyotrophic lateral sclerosis (ALS).^64^, ^68^, ^69^ Therefore, the cGAS‐STING signaling pathway serves as an important target for the treatment of NDs.

### 4.1 Ischemic stroke

Ischemic stroke is an acute cerebrovascular disease that damages brain tissue due to sudden blockage of blood vessels in the brain that prevents blood from flowing into the brain. In recent years, emerging evidence suggests that the pathophysiology of ischemic stroke includes oxidative stress, cellular excitotoxicity, neuroinflammation, and cell death processes. A substantial body of research has documented that in preclinical stroke models, the activation of cGAS‐STING signaling pathway exacerbates brain damage. Consistently blocking this pathway has been shown to significantly improve functional outcomes after stroke.

After ischemic injury, brain tissue releases a substantial amount of DNA, which is engulfed by the surrounding microglia, thereby initiating the cascade of the cGAS‐STING signaling pathway.^70^ Following ischemic events, there is an observed increase in the co‐localization of cGAS and STING, predominantly within microglia. Additionally, elevated levels of cGAS and STING expression in microglia have also been documented.^71^, ^72^, ^73^ Activation of cGAS‐STING pathway is associated with proinflammatory polarization of microglia. Ling et al. demonstrated that in ischemic stroke, cGAS‐STING regulates IRF3 and NF‐κB to promote microglial polarization toward the M1 phenotype and inhibit microglial polarization of M2, thus causing neuroinflammation (TNF‐α, IL‐6, IL‐1β) and cognitive function. In contrast, inhibition of STING by C‐176 alleviates cognitive impairment.^72^ Furthermore, the knockdown of cGAS in the cGAS‐STING signaling pathway was found to promote microglial M2 polarization and improve neuroinflammation and cognitive function in ischemic stroke.^73^ Rui et al. showed that the upregulated cGAS‐STING axis leads to neuroinflammation and microglial pyroptosis through NF‐κB and NLRP3 following an animal model of cerebral venous sinus thrombosis (CVST), inhibition of cGAS or downregulation of STING ameliorates neuroinflammation and microglial pyroptosis, thus improving ischemic stroke.^74^ The protective mechanism of neuroprotective factors after stroke has also been reported to be related to the cGAS‐STING pathway. Administration of exogenous Activin A prior to transient focal ischemia or after reperfusion can reduce neuronal ischemic damage by inhibiting cGAS‐STING‐mediated excessive autophagy.^75^

### 4.2 Alzheimer's disease

Alzheimer's disease (AD) is the most common NDs and accounts for approximately 60%–70% of dementia patients. The pathogenesis of AD remains unclear, leading to therapeutic approaches that only utilize symptomatic strategies rather than preventive strategies. Currently, the mechanisms of the amyloid and tau hypothesis are the most widely accepted in AD. However, these hypotheses cannot fully explain neuronal degeneration in AD. Recent evidence finds that neuroinflammation plays an important role in AD pathology.^76^ Biomarkers of AD‐related neuroinflammation include NF‐κB and cGAS‐STING pathways.

Larrick et al. showed that cGAS‐STING is elevated in AD mouse brain and human AD fibroblasts.^68^, ^77^ The activation of the cGAS‐STING pathway may be associated with oxidative damage of mtDNA and DNA double‐strand breaks caused by toxic proteins.^78^, ^79^ In a recent study, the binding of dsDNA and cGAS was directly identified in 5xFAD mouse brain tissue. Compared with control group, the interaction between cGAS and dsDNA increased by more than four times.^80^ The cognitive impairment, amyloid‐beta pathology, neuroinflammation, and other sequelae associated with AD were significantly attenuated in 5xFAD mice lacking cGAS.^80^The activation of cGAS‐sting appears to be associated with the phenotypic transformation of astrocytes, as the deficiency of cGAS in microglia inhibits the neurotoxic A1 astrocyte phenotype.^80^ The mechanism of exogenous nicotinamide riboside (NR) supplementation to improve AD pathology is also related to the cGAS‐STING pathway. Hou et al. demonstrated that NR reduces neuroinflammation and cell senescence and improves cognitive function in AD mice by regulating the cGAS‐STING pathway.^81^ However, the role of this pathway in the pathological process of AD remains a subject of controversy. Several studies have demonstrated that intravenous administration of the STING activator cGAMP can significantly ameliorate cognitive deficits and AD pathology in APP/PS1 transgenic mice by inducing TREM2 expression.^82^ This further underscores the intricate involvement of the cGAS‐STING pathway in the pathological progression of AD.

### 4.3 Parkinson's disease

Parkinson's disease (PD) is a progressive disorder that affects the nervous system and parts of the body controlled by the nerves. The main pathological change in PD is the degeneration and death of dopaminergic neurons in the substantia nigra area, which leads to a decrease in dopamine content in the ventral striatum and the dysfunction of motor regulation and cognitive impairment. Dopamine (DA), dopaminergic neuron cell death, oxidative stress, and neuroinflammation have been shown to play significant roles in the pathogenesis of PD.^83^, ^84^ The cGAS‐STING pathway is most likely to be activated during the progression of PD, as injured dopaminergic neurons can produce dsDNA. Some experimental data have been supporting this theory in recent years. The accumulation and misfolding of α‐synuclein (α‐syn) protein is a characteristic pathological feature in postmortem tissue of human PD postmortem tissue.^85^, ^86^ Direct injection of α‐syn preformed fibrils (PFFs) into the striatum of mice is a method to create PD models.^87^ The PFFs induce neuroinflammation through the cGAS‐STING pathway in the microglia of PD mice. In a PFF‐induced PD model, STING^gt^ mice were found to reduce neuroinflammation and protect from neurodegeneration.^86^ These phenomena were also observed in MPTP‐induced neurotoxic PD models.^88^ Further investigation revealed that the cGAS‐STING pathway is activated not only in microglia but also in astrocytes. In the pathological conditions of PD, STING expression is upregulated in astrocytes, leading to increased binding with the transcription factor YY1. This interaction prevents nuclear translocation of YY1 and subsequently enhances the expression of Lipocalin‐2. Moreover, activation of this axis within astrocytes directly correlates with neurodegeneration.^89^ These data showed that cGAS‐STING‐driven neuroinflammation plays a vital role in the mechanism of neurodegeneration in PD. Inhibitors of serum/ glucocorticoid‐related kinase 1 (SGK1) protect midbrain dopamine neurons by inhibiting neuroinflammation regulated by the STING pathway while improving pathological synuclein alpha (SNCA) aggregation and behavioral deficits related to PD in in‐vitro and in‐vivo PD models.^90^

### 4.4 Huntington's disease

Huntington's disease (HD), also known as significant chorea or Huntington's chorea, is an autosomal dominant neurodegenerative disease. The prevalence of HD is approximately 4–12 per 100,000 people in the United States and Europe.^91^ Generally, the onset of the disease occurs in middle age. The main symptoms of HD include involuntary dance movements, cognitive impairment, and behavioral changes.^92^ The leading cause is the mutation of the Huntington gene on the patient's fourth chromosome, which produces a mutated protein that gradually aggregates in cells to form large molecular clusters that accumulate in the brain and affect the function of neuronal cells in the striatum area of the brain.^93^ Previous studies have demonstrated that the pathogenesis of HD includes mitochondrial toxicity, mitochondrial toxicity, inflammation, oxidative stress, neurotransmitter disturbance, and transcriptional dysregulation, eventually leading to neuronal death.^94^ Nowadays, increasing evidence indicates that HD is associated with the cGAS‐STING pathway. Kai et al. demonstrated that mitochondrial cytosolic DNA could activate the cGAS‐STING pathway to induce inflammatory responses in the striatum of postmortem HD patients.^68^

Furthermore, Abhishek J found that HD mice had increased mtDNA release, which activated the cGAS‐STING‐IRF3 pathway and the generation of inflammatory cytokine generation; in an HD cell culture model, transfection of DNaseI into these cells reduced inflammation.^95^ In the same study, Sharma et al. reported that the cGAS‐STING pathway is important in regulating inflammatory responses in HD. However, depletion of cGAS reduced the activity of the cGAS‐STING pathway and decreased the expression of inflammatory genes in the cells model of HD.^96^

### 4.5 Amyotrophic lateral sclerosis

**FIGURE 1:**
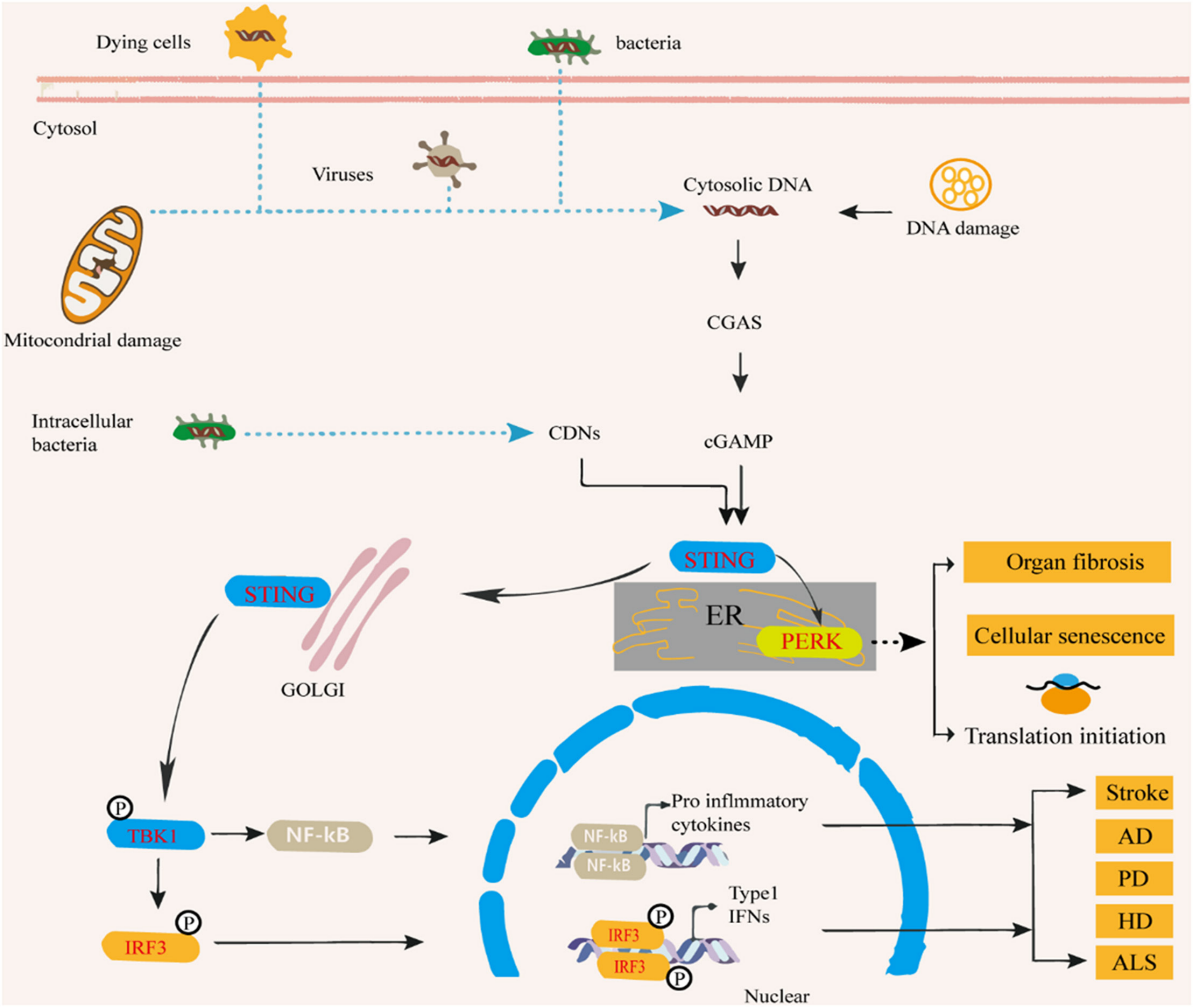
The canonical cGAS‐STING pathway model produces neuroinflammation in ND. The non‐canonical cGAS‐STING pathway can regulate the control of innate immunity of cap‐dependent messenger RNA translation and cell senescence or organ fibrosis.

Amyotrophic lateral sclerosis (ALS) is one of the most severe degenerative diseases of the central nervous system (CNS). ALS is also called motor neuron disease (MND). It occurs after damage to the upper and lower motor neurons, resulting in atrophy and cognitive/behavioral dysfunctions.^97^, ^98^ Currently, the mechanism of ALS is unclear, but several studies suggest that impaired RNA metabolism, mitochondrial dysfunction, neuroinflammation, and neuronal death are involved in disease pathogenesis.^99^ For example, in ALS, the misfolded SOD1 protein causes mitochondrial damage. The released mtDNA and RNA activate the IRF3 and type I interferon (IFN‐I) genes regulated by the cGAS‐STING pathway, leading to a massive inflammatory response.^100^ In addition, in ALS, reactive oxygen species (ROS) in neurons cause mitochondrial damage and release mtDNA. The mtDNA acts on microglia and astrocytes around neurons to produce an inflammatory response through the cGAS‐STING pathway.^101^ Notably, Van Damme P found that the TAR DNA‐binding protein (TDP‐43) protein is increased in the central nervous system of most ALS patients. TDP‐43 was found to induce mitochondrial damage and release mtRNA in an animal model of ALS. TDP‐43 was found to induce mitochondrial damage and release mtRNA in an ALS animal model. Next, mtRNA activates an inflammatory response that involves type I interferon and NF‐κB signaling through the cGAS‐STING pathway.^102^ However, genetic deletion of STING improves the ALS mouse model.^103^ Similarly, STING inhibitors suppress the elevation of type I IFN in ALS patients.^104^ (Figure 1).

## 5 INHIBITORS OF cGAS‐STING SIGNALING PATHWAY

In recent years, several studies have reported that anti‐inflammatory therapies can protect against NDs, suggesting that neuroinflammation plays an essential role in NDs.^68^ For most NDs that develop with elevated levels of neuroinflammation and increased pro‐inflammatory cytokines, inhibition of the cGAS‐STING pathway reduces neuroinflammation and improves NDs, and this pathway is expected to be a key target for NDs therapy. The development of inhibitors targeting this pathway has currently experienced significant advancements.

### 5.1 cGAS‐targeted inhibitors

As the initiator of the cGAS‐STING signaling pathway, inhibiting its activity can prevent activation of the downstream pathway and may play a role in the treatment of NDs. Inhibitors affect cGAS activation mainly by occupying the cGAS active site or blocking cGAS binding to dsDNA. In addition, some inhibitors with unknown mechanisms are equally capable of affecting cGAS activity as well.

Hall et al. adopted NMR screening of fragment libraries and continued to optimize the results of previous studies to obtain a high‐affinity inhibitor PF‐06928215, which could bind to and inhibit the active site of cGAS, but did not inhibit the induction of IFN and lacked cellular activity.^105^ RU.521 reduces the affinity of cGAS for ATP and GTP, inhibits 2′3′‐cGAMP production, and decreases the level of IFN‐β.^106^ Zhao et al. established the high‐resolution (1.8 A) crystal structure of the catalytic domain hcGAS based on the study of PF‐06928215, which laid a solid foundation for the development of subsequent cGAS inhibitors. For the first time, the team used virtual screening and thermal shift analysis to obtain catalytic structural domain inhibitors of cGAS and PPiase‐coupled assay to identify cGAS inhibitors S3 with similar potency to PF‐06928215 and RU.521.^107^ Methylpyrazole (G140) or 2‐aminopyridine (G150), reported by Lama, have the exact mechanism as described above and were able to show inhibition of cGAS in THP1 cells and human macrophages.^108^ At the same time, Naringenin, which is often used as an antioxidant and free radical scavenger, was also able to bind to the active site of cGAS, reducing the mRNA levels of IL‐1β, IL‐6, NF‐κB, and IL‐8 in LX2 cells, and also dose‐dependently reducing the expression of IRF3 protein.^109^

Steinhagen first proposed the cGAS inhibitor A151, which is capable of blocking self‐DNA and suppressive oligodeoxynucleotides containing repetitive TTAGGG motifs in mammalian telomeres,^110^ is dependent on telomeric sequences and phosphorothioates and inhibits the production of type I IFN by human monocytes through competing with DNA to prevent cGAS activation.^111^ Suramin was also able to replace DNA bound to cGAS and inhibit the activity of the cGAS enzyme, while the addition of suramin to THP1 cells was shown to reduce the levels of IFN‐β mRNA and protein.^112^ An's group identified several antimalarial drugs that interacted with the cGAS/dsDNA complex using a mouse cGAS/DNA target (PDB 4LEZ) screen, hydroxychloroquine (HCQ) and quinacrine (QC) inhibit IFN‐β production by inserting into the cGAS‐dsDNA binding groove and blocking the binding of dsDNA to cGAS.^113^ Based on the above, this team then reported a new aminoacridine antimalarial analog, X6, which significantly reduced in cGAMP, IFN‐β, and interferon‐stimulated genes (ISG) in Trex1^−/−^ mice compared to the first‐generation inhibitors.^114^ Aspirin also inhibits cGAS by acetylating the cGAS residues Lys384, Lys394, or Lys414, blocking the binding of cGAS to DNA and effectively preventing cGAS‐mediated immune responses.^115^

In particular, there are inhibitors of unknown or undisclosed mechanisms that also inhibit cGAS activity. Specifically, a virtual screen of potentially druggable pockets around Lys347 and Lys394 in h‐cGAS and the only mechanistically active protein–protein interface, z918, of the cGAS dimer itself. CU‐76 had higher cellular activity and was shown to selectively inhibit the DNA pathway in human cells. It is possible that the inhibitor may bind to a groove next to the zinc loop, inhibiting cGAS dimerization through conformational changes, and CU‐76 and its analogs may bind to different pockets. However, more precise mechanisms must be further determined by co‐crystallization studies.^116^ Compound C (Dorsomorphin) has the ability to reduce interferon expression by preventing cGAMP accumulation and has also been shown to rescue the autoimmune phenotype in a mouse model deficient in the Trex1 gene, as verified by in vivo assays. However, compound C does not bind directly to the active site of cGAS, it is suggested that cGAS‐mediated activity may be inhibited by suppressing some upstream genes.^117^ Fluvoxamine suppresses cyclic GMP‐AMP synthase (cGAS) and interferon gene stimulating factor (STING) and inhibits the activation of downstream targets, including PERK/eIF2α/c‐Myc/miR‐9‐5p/TBPL1 and TBK1 / YAP / JNK1/2 / Bnip3 / CaMKII / cofilin signaling, which reduces the expression of inflammatory factors, although the mechanism of inhibition is still unclear.^118^ (Table 2).

**TABLE 2:** Inhibitors targeting cGAS.

Inhibitors		Mechanism	Model	Reference
cGAS	PF‐06928215	High affinity to the cGAS catalytic site, but unable to inhibit IFN induction and lack of cellular activity	THP‐1 cells	Hall et al. (2017)
	RU.521	Binding to the active site of cGAS, inhibiting 2′3′‐cGAMP production and reduces IFN‐β levels	BMDMs in Trex1−/− mice	Vincent et al. (2017)
	G140	Occupying the ATP and GTP binding active site and inhibiting THP‐1 cell activity	THP‐1 cells and human macrophages	Lama et al. (2019)
	S3	Binding to the active site of cGAS	_	Zhao et al. (2020)
	Naringenin	Binding to the active site of cGAS and reducing mRNA levels of IL1β, IL6, NF‐κB and IL8	LX2 cells	Chen et al. (2023)
	HCQ	Insertion of the cGAS‐dsDNA binding groove inhibiting the production of IFN‐β	THP‐1 cells	An et al. (2015)
	X6	Significant reduction in cGAMP, IFN‐β, interferon‐stimulated genes	Trex1−/− mice	An et al. (2018)
	A151	Blocking cGAS activation by competing with DNA	THP‐1 cells、Trex1−/− mice	Steinhagen et al. (2018)
	Suramin	Replacing DNA bound to cGAS and inhibiting cGAS enzyme activity reduces IFN‐β mRNA and protein levels	THP‐1cells	Wang et al. (2018)
	Aspirin	Acetylation of cGAS residues Lys384, Lys394 or Lys414 prevents the binding of cGAS to DNA	AGS patient cells and AGS mouse model	Dai et al. (2019)
	Compound C	cGAS activity is inhibited by repression of some unknown upstream gene.	Trex1−/− mice	Lai et al. (2020)
	Fluvoxamine	Inhibits activation of downstream targets, including PERK/eIF2α/c‐Myc/miR‐9‐5p/TBPL1 and TBK1/YAP/JNK1/2/Bnip3/CaMKII/cofilin signaling	Lung fibrosis mouse model	Xie et al. (2023)

### 5.2 STING‐targeted inhibitors

STING is the convergence site of the DNA sensor, which can further transmit downstream activation signals. Targeted STING inhibitors are divided into covalent inhibitors and competitive inhibitors of the CDN binding site.

In Trex1^−/−^ mice and murine BMDMs, C‐176, and C‐178 are capable of forming covalent bonds with the Cys91 STING protein and inhibit the palmitoylation of the STING protein from interfering with the interaction of STING with TBK1. On the basis of this study, H‐151 was further obtained by structural optimization. It has significant inhibitory activity in human cells and in vivo by covalently binding to Cys91 to block STING activation‐induced palmitoylation, leading to abrogation of type I IFN responses and reduction of TBK1 phosphorylation.^119^ It has been found that NO2‐FAs, an endogenous substance produced by adding nitrogen dioxide (NO_2_) to unsaturated fatty acids during virus infection, have been found to modify STING covalently by Michael addition reaction to adjacent cysteines at positions 88 and 91 or N‐terminal histidine, thereby inhibiting STING palmitoylation and type I IFN in host cells.^120^ Cellular lipid peroxidation specifically inhibits cGAS‐STING signaling through its final products, such as 4‐hydroxynonenal (4‐HNE), which selectively modulates STING signaling through its enzymatic activity and lipid peroxidation by inactivation of GPX4, leading to carbonylation of STING at C88 and inhibiting its translocation from the endoplasmic reticulum to the Golgi complex, and the terminal product of lipid peroxidation 4‐HNE inhibits HSV‐1 infection induced IFN‐β expression.^121^

Li et al. isolated the cyclic peptide Astin C from the medicinal plant Aster tataricus, which specifically bound to the C‐terminal activation site of STING and consequently inhibited IRF3 recruitment, blocking downstream signaling in the cGAS‐STING pathway, thereby inhibiting the innate inflammatory response triggered by cytoplasmic DNA.^122^ Compound SN‐011 is a highly effective inhibitor of STING that binds to the cyclic dinucleotide (CDN) binding pocket of STING with greater affinity than endogenous 2′3′‐cGAMP blocking CDN conjugation while locking the STING dimer in an open inactive conformation and inhibiting interferon and inflammatory cytokine expression.^123^ Siu used the symmetry of the CDN‐binding domain to design Compound 18, a small molecule inhibitor that binds to the STING protein and inhibits IFN‐β production by binding to the 2′3′‐cGAMP binding site.^124^ (Table 3).

**TABLE 3:** Inhibitors targeting STING.

Inhibitors		Mechanism	Model	Reference
STING	C176/ C178	Covalent bonding with Cys91 inhibits palmitoylation of STING protein	Trex1−/− mice	Haag et al. (2018)
	H‐151	Covalent bonding with Cys91 inhibits palmitoylation of STING protein	HEK293T cells Trex1−/− mice	Haag et al. (2018)
	NO2‐FAs	Michael addition reaction with Cys88/91 or His16 to inhibit STING palmitoylation	THP‐1 cells and BMMs	Hansen et al. (2018)
	4‐HNE	Inhibiting STING translocation from the ER to the Golgi	THP‐1 cells	Jia et al. (2020)
	Astin C	Binding to the C‐terminal activation site of STING to inhibit IRF3 recruitment	Trex1−/− mice	Li et al. (2018)
	SN‐011	Binding to the CDN binding pocket of STING	Trex1−/− mice	Hong et al. (2021)
	Compound18	Binding to the STING active site and inhibiting IFN‐β production	THP‐1cells	Siu et al. (2019)

## 6 THERAPEUTIC POTENTIAL OF cGAS‐STING INHIBITORS IN NDs

A substantial body of evidence demonstrates the protective effects of immunotherapy on neuroinflammation and neurodegeneration in various diseases of the nervous system. The identification of the cGAS‐STING pathway offers a novel avenue for this therapeutic approach. However, there is currently limited research on the application of inhibitors targeting the cGAS‐STING pathway in the CNS, and these studies are still at the pre‐clinical research stage. Nevertheless, investigations utilizing cell culture models and animal models have shown that cGAS‐STING inhibitors effectively mitigate NDs' pathology, holding significant promise for the development of new drugs.

Inhibitors that have demonstrated efficacy in animal models of NDs include RU.521, A151, C176, and H‐151. Recent studies have shown that RU.521 and A151 effectively reduce infarct volume, and improve behavioral outcomes.^80^ C176 inhibits the phosphorylation of STING and has been reported to improve prognosis of both ischemic stroke and PD. Administration of C176 via intraperitoneal route 30 min after MCAO can enhance hippocampal neurocognitive function.^72^ Moreover, researchers have developed a new nano‐drug to enhance the therapeutic impact of stroke by combining the DNase‐memetic Ce4+ enzyme and C176 synergistically.^125^ Similarly, C‐176 treatment demonstrates efficacy in PD models by ameliorating MPTP‐induced dopaminergic neurotoxicity and motor deficits. H151 shares a similar mechanism with C176 and has been found to reduce neuroinflammation and pathological protein deposition in ALS and AD mice.^80^

There are still numerous challenges associated with the clinical application of these inhibitors. First, due to the complexity of the cGAS‐STING pathway, it involves in various physiological processes within the body, exhibiting multiple effects. Therefore, careful attention must be given to ensure the safety of inhibiting neuroinflammation through targeting this pathway. From an immunological perspective, such inhibition may potentially increase the risk of secondary infections. However, since this pathway is upstream of type I IFN, inhibitors toward the cGAS‐STING pathway might cause less harm to host defenses compared to directly blocking type I IFN. Second, the precise timing for activation of the cGAS‐STING pathway in different NDs remains unclear. This uncertainty poses a challenge when establishing an optimal time window for applying inhibitors that can effectively exert their protective effects while minimizing toxicity risks. Lastly, another significant hurdle in drug development lies in addressing the diversity of STING pathways. This diversity manifests as high heterogeneity among human populations and variations in structure and signaling between different species.^126^ Multiple alleles encoding STING have been reported across diverse ethnic groups among humans, while differential expression of STING protein have been identified based on cell genotypes.^127^ Furthermore, disparities exist between mice and humans regarding STING protein structure which has hindered the successful clinical translation of STING agonist DMXAA for anti‐tumor purposes.^126^ Overcoming these challenges holds great significance in advancing drug development targeting cGAS‐STING.

## 7 CONCLUSIONS AND FUTURE PERSPECTIVES

This review summarized the mechanisms by which the cGAS‐STING pathway generates neuroinflammation in NDs and the target inhibitors. It is expected that understanding the mechanisms of the cGAS‐STING signaling pathway in the nervous system will lead to a thorough understanding of the critical role of the pathway in NDs. cGAS‐STING signaling plays a significant part in the innate immune response. Its abnormal activation is strongly associated with various autoimmune diseases and as a therapeutic target in inflammatory diseases.^128^ Using a mouse model of MPTP‐induced neurotoxic PD and knocking out cGAS wild‐type adult male mice (cGAS^−/−^), Ma's team concluded that the deficiency of cGAS in microglia controls MPTP‐induced neuroinflammation and neurotoxicity to some extent, providing support for inhibition of cGAS as a therapeutic target.^20^ Similarly, Hinkle's study confirmed that knockout STING is neuroprotective in the α‐Syn preformed fibril (α‐Syn‐PFF) model and that activation of cGAS/STING may exacerbate pathological neuroinflammation in α‐synuclein diseases such as inflammatory PD,^85^ and suggest that STING inhibition may be therapeutic in idiopathic PD and possibly other human α‐syn. We hypothesize that inhibition of the typical cGAS‐STING pathway reduces neuroinflammation and improves NDs and that rational drug therapy targeting the cGAS‐STING pathway may be an effective therapeutic strategy for NDs.

## AUTHOR CONTRIBUTIONS

XFG, LY, JWW, and YW initiated the idea and wrote the manuscript. YL, LXD, and LL consulted literature. ZPF and XJZ revised the manuscript.

## CONFLICT OF INTEREST STATEMENT

The authors declare that they have no potential competing interests.

## Data Availability

Data sharing not applicable to this article as no datasets were generated or analysed during the current study.
